# Many Good Reasons to Switch from Vitamin K Antagonists to Non-Vitamin K Antagonists in Patients with Non-Valvular Atrial Fibrillation

**DOI:** 10.3390/jcm10132866

**Published:** 2021-06-28

**Authors:** Giovanni Luca Botto, Pietro Ameri, Raffaele De Caterina

**Affiliations:** 1 Department of Cardiology—Electrophysiology, ASST Rhodense, Garbagnate Milanese, 20024 Milan, Italy; gbotto@asst-rhodense.it or; 2 Cardiovascular Disease Unit, IRCCS Ospedale Policlinico San Martino, IRCCS Italian Cardiology Network, 16132 Genova, Italy; 3 Department of Internal Medicine, University of Genova, 16132 Genova, Italy; 4 Division of Cardiology, Department of Surgical, Medical and Molecular Pathology and Critical Care Medicine, University of Pisa, 56124 Pisa, Italy; raffaele.decaterina@unipi.it; 5 Fondazione Villa Serena per la Ricerca, Città Sant’Angelo, 65103 Pescara, Italy

**Keywords:** anticoagulant, vitamin K, atrial fibrillation, cardioembolism, stroke

## Abstract

Non-vitamin K oral anticoagulants (NOACs) are the first choice for prophylaxis of cardioembolism in patients with non-valvular atrial fibrillation (AF) who are anticoagulant-naïve, as well as the preferable anticoagulation strategy in those who are on vitamin K antagonists (VKAs), but with a low time in therapeutic range (TTR). Nonetheless, there are many good reasons to consider switching from VKAs to NOACs also when TTR is >70%. From the pharmacological standpoint, anticoagulation with VKAs may remain erratic even in those patients who have high TTR values, owing to the mode of action of this drug class. Furthermore, experimental data suggest that, unlike VKAs, NOACs favorably modulate the effects of factor Xa and thrombin in the cardiovascular system through the protease-activated receptor family. Clinically, the most striking advantage provided by NOACs over VKAs, irrespective of the TTR, is the substantially lower risk of intracranial hemorrhage. NOACs have also been associated with less deterioration of renal function as compared with VKAs and may confer protection against cardiovascular events not strictly related to AF, especially the acute complications of peripheral artery disease. In this narrative review, we discuss the evidence according to which it is warranted to systematically substitute NOACs for VKAs for the prevention of AF-related stroke and systemic embolism.

## 1. Introduction

Non-valvular atrial fibrillation (AF) is the most common arrhythmia encountered in clinical practice; worldwide, 43.6 million individuals had prevalent AF or atrial flutter in 2016 [1].

The currently estimated prevalence of AF in adults is 2–4% [1], and a constant increase is expected, with extended longevity in the general population and intensifying search for undiagnosed AF [2]. Therefore, it is estimated that the number of patients with AF will double over the next 40 years [3].

AF is associated with substantial morbidity and mortality, thus giving a significant burden to patients and the health care system. One of the most serious complications of AF is a thromboembolism, mainly stroke [4], which occurs regardless of the presence or absence of symptoms [5]. The risk of thromboembolism correlates with increases in the CHADS_2_ and CHA_2_DS_2_-VASc scores [6,7,8].

Vitamin K antagonists (VKAs) were the only oral anticoagulants (OACs) available for the prophylaxis of AF-related thromboembolism until 2009 [9]. VKAs are strongly effective in preventing thromboembolism in patients with AF. In a meta-analysis of six phase III randomized controlled trials (RCTs), adjusted-dose warfarin, compared with placebo/no treatment, reduced the relative risk of stroke by 64%, and the risk of all-cause mortality by 26% [10].

However, VKA therapy has several limitations, including the need of overlapping heparin until the targeted effect is achieved due to its slow onset/offset of action, significant food and drug interactions, narrow therapeutic window, and unpredictable anticoagulant effect, making it difficult to implement this type of OAC in routine clinical practice [11]. Moreover, VKA therapy requires routine international normalized ratio (INR) monitoring, and frequent dose adjustments, resulting in significant inconvenience and risk. Warfarin was responsible for most emergency room access and emergency hospitalizations for recognized adverse drug events in older adults (≥65 years of age) in the US from 2007 through 2009 [12].

This explains the poor utilization and the high discontinuation rate of warfarin in the real world, as well as the inadequate level of anticoagulation reached in many patients [13].

Non-vitamin K oral anticoagulants (NOACs) are a class of OACs that directly inhibit thrombin (dabigatran) or activated factor X (FXa; apixaban, edoxaban, and rivaroxaban). In a large meta-analysis of four RCTs including almost 72,000 patients with AF, these drugs were compared to warfarin and showed a significant reduction in the primary endpoint of stroke/systemic embolism (SE) by 19% and in all-cause mortality by 10%. Additionally, NOACs provide a significant relative reduction in intracranial hemorrhage (ICH) by 52% with similar major bleeding compared to warfarin [14]. Similar to VKAs, the efficacy of NOACs is consistent across types of AF (paroxysmal, persistent, and permanent) [15].

NOACs were introduced to circumvent the limitations associated with VKA therapy in clinical practice, since they have a more predictable response, rapid onset/offset of action, no interaction with food, and fewer drug–drug interactions. These pharmacokinetic properties enable the administration of fixed doses without the need for routine coagulation monitoring, thereby simplifying treatment. Nevertheless, the quantification of NOAC concentration and anticoagulant effect may be needed in emergency situations, such as serious bleeding and need for urgent surgery, or special situations, such as patients with extreme body weight, potential major drug–drug interactions, or suspected overdosing. The presence of dabigatran or FXa inhibitors can be assessed qualitatively by means of the activated partial thromboplastin time and prothrombin time, respectively, but these parameters do not allow gauging NOAC levels and activity. The anticoagulant effect of NOACs can be instead quantified with the diluted thrombin time and ecarin chromogenic assay for dabigatran and the chromogenic anti-FXa assay for FXa inhibitors. However, these quantitative tests require adequate laboratory expertise and should not be used routinely [16].

The European Society of Cardiology (ESC)/European Association for Cardio-Thoracic Surgery (EACTS) guidelines favor the use of NOACs over VKAs for stroke prevention in OAC-naïve patients with AF, with a class 1A recommendation [9].

Although NOACs are particularly attractive for AF patients starting anticoagulation therapy, it is not clear whether AF patients who are well anticoagulated on VKA should switch to a NOAC.

The National Cardiovascular Disease Registry’s (NCDR) Practice Innovation and Clinical Excellence (PINNACLE) registry longitudinally follows patients with AF, providing insights into patterns of switching OAC therapy and into patient and practice-level factors associated with switching [17]. Among 383,008 AF patients initially prescribed warfarin, 16.3% switched to NOACs, and 14.8% discontinued anticoagulation. Switched patients were significantly more likely to be young, women, white, and have private insurance. Switching was less likely with increased stroke risk (OR 0.92, 95%CI 0.91–0.93 per 1-point increase CHA2DS2-VASc), but more likely with increased bleeding risk (OR 1.12, 95%CI 1.10–1.13 per 1-point increase HAS-BLED) [18]. Potential explanations for the relatively low rate of switching include therapeutic inertia, where physicians may choose not to change the type of OAC if patients have been stable on VKAs without adverse events, in the absence of a specific clinical issue. However, increased cost and other barriers to switching must also be addressed, including patient perspectives and preferences.

Regardless of the explanation, the low rate of switching from VKAs to NOACs represents an important target for NOAC implementation [19].

**Figure 1:**
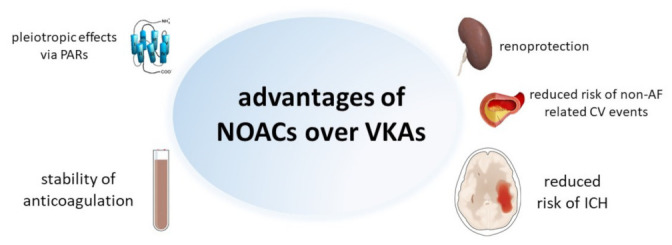
Pharmacological and clinical aspects supporting the use of NOACs over VKAs in patients with AF. AF: atrial fibrillation; ICH: intracranial hemorrhage; PARs: protease-activated receptors. The pictures within the Figure were obtained from Wikimedia Commons and do not have copyright.

Here, we review the many reasons by which it is advisable to substitute NOACs for VKAs, ranging from pharmacological advantages to clinical benefits (Figure 1).

## 2. Pharmacological Considerations Supporting the Switch from Vitamin K Antagonists to Non-Vitamin K Antagonists

### 2.1. Instability of Anticoagulation with Vitamin K Antagonists

The quality of anticoagulation on VKA therapy can be measured as time in the therapeutic range (TTR) [20]. In patients receiving VKAs, the TTR is the percentage of days in a row when the INR is within the therapeutic range (usually considered to be 2.0 to 3.0). It is intuitive that patients who are rarely in the therapeutic range receive little or no benefit from the treatment with VKAs. Several reports have indicated an association between low TTR and an increase in stroke/SE and major bleeding in patients on VKAs [21,22,23]. Additionally, secondary analyses of RCTs with NOACs demonstrated that warfarin with high TTR values (usually ≥70%) had comparable efficacy and safety to those of NOACs [24,25,26]. Therefore, it is suggested that providers can consider continuing VKA if patients are able to maintain a TTR on VKA ≥70% [19], obviously assuming that patients receiving VKA who have stable INR values remain stable over time.

**Figure 2:**
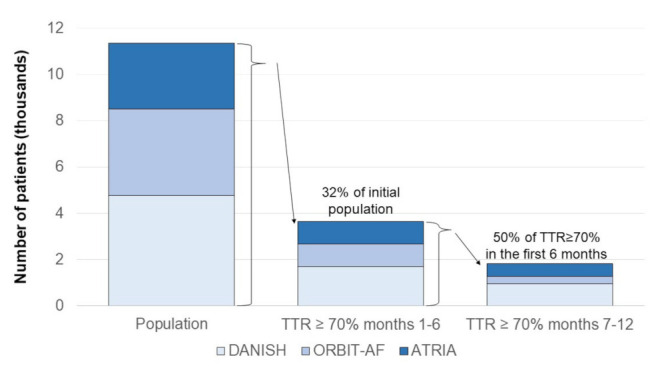
Proportion of AF patients taking warfarin and with persistently satisfactory anticoagulation in warfarin in three large registries. The figure was drawn based on the published data in references [27,28,29]. TTR: time in therapeutic range.

However, limited data are available to support this claim, since studies suggest that less than one-third of patients on warfarin achieve an initial TTR ≥70%, and nearly half of them continue to have a TTR ≥70% over the following months [27] (Figure 2).

Among 3749 patients taking warfarin (mean age, 75 years), included in the Outcomes Registry for Better Informed Treatment of Atrial Fibrillation (ORBIT-AF), only 968 (26%) had 80% or more of INR values in the 2.0–3.0 range during the first six months. Of the patients with stable INR during the first six months, only 34% (95% CI, 31–37%) remained stable over the subsequent year [27].

In the community-based Anticoagulation and Risk Factors in AF (ATRIA) cohort, 987/2841 (35%) patients with TTR ≥70% in an initial 6-month period were identified. Among them, 57% persisted with TTR ≥70% and 16% deteriorated to TTR <50% in the following six months. Only an initial TTR ≥90% independently predicted TTR ≥70% in the following six months (adjusted OR 1.47, 95% CI 1.07–2.01). Heart failure was moderately associated with TTR <50% (adjusted OR 1.45, 95% CI 1.00–2.10) [28].

Finally, an analysis of the data from the Danish nationwide registries identified 4772 AF patients still on VKA six months after initiation, of whom 1691 (35%) had a TTR ≥70%, and 3081 (65.6%) had a TTR <70%. Among patients with TTR ≥70% at baseline and still on OAC treatment 12 months after inclusion in the study, only 513 (55.7%) still had a TTR ≥70%. Moreover, compared with prior TTR ≥70%, prior TTR <70% was not associated with a higher risk of stroke/thromboembolism (HR 1.14, 95% CI 0.77–1.70) or major bleeding (HR 1.12, 95% CI 0.84–1.49) [30]. By contrast, the outcome of patients with initial TTR ≥70% was better than the one of patients with TTR <70% when reallocation from a TTR group to another during follow-up was taken into account [29], confirming that TTR fluctuations over time are clinically relevant.

A common belief has been that the patients with stable INR while taking VKAs would continue to be stable and derive less benefit from switching to NOACs [19,30]. However, instability of anticoagulation intensity is common among patients with AF treated with VKAs, even when anticoagulation control has been satisfactory in the past. Moreover, since VKA stability is difficult to predict, patients who have done well taking VKAs should not deter physicians from recommending the substitution of a target-specific anticoagulant (NOAC).

### 2.2. Availability of an Antidote

All OACs can be counteracted by antidotes: vitamin K for VKAs, idarucizumab for dabigatran, and andexanet alfa for FXa inhibitors [9,16]. No head-to-head study has compared the efficacy of these agents; hence their availability cannot be viewed as a reason to prefer one class of OAC over another. Nonetheless, in the emergency setting with the need for prompt restoration of coagulation, the rapid action of NOAC antidotes, especially idarucizumab, may be advantageous as compared with the slower one of vitamin K.

### 2.3. Periprocedural Oral Anticoagulation

In four open-label controlled trials investigating the best OAC strategy in subjects undergoing left atrial catheter ablation of AF, the rates of bleeding were lower or comparable with uninterrupted NOACs than with uninterrupted warfarin with target INR 2.0–3.0 [31,32,33,34]. The safety of NOACs in this setting has also been demonstrated in real-life patients [35,36]. Thus, NOACs are a better alternative to well-managed VKAs when catheter ablation of AF is planned. Moreover, for BID-dosed NOACs, omission of the dose in the evening before the procedure (“minimally interrupted strategy”) may be as safe and effective as administration of the last dose in the morning of the day of the procedure (“truly uninterrupted strategy”) [16].

NOACs have also been compared with VKAs as part of the antithrombotic therapy for concomitant AF and percutaneous coronary intervention. On aggregate, the combination of a NOAC plus a P2Y_12_ inhibitor is associated with fewer bleeding episodes than a therapeutic regimen including a VKA and one or two antiplatelet agents [37]; however, dropping aspirin and using only a NOAC and a P2Y_12_ inhibitor may imply a higher risk of coronary events [38].

### 2.4. Effects Beyond Coagulation

FXa and thrombin are central to the coagulation process, but also act on several cell types via protease-activated receptors (PARs) [39]. By binding and cleaving these transmembrane G protein-coupled receptors, FXa and thrombin initiate downstream intracellular signaling pathways that, in the cardiovascular (CV) system, are protective or detrimental depending on the context, the local concentrations of coagulation factors, and the extent of PAR engagement. While in physiological conditions coagulation proteases exert vasculoprotective effects through PARs, in pathological conditions they elicit PAR-driven cellular events that promote endothelial dysfunction, vascular muscle cell impairment, and inflammation [40]. Along with thrombomodulin and endothelial protein C receptor, thrombin also participates in the activation of a specific PAR, PAR-1, by activated protein C, which is anticoagulant, anti-inflammatory, and antiapoptotic [41].

By directly targeting FXa and thrombin, NOACs better target the PAR-mediated harmful actions of these coagulation factors [40]. Furthermore, VKAs inhibit the synthesis of protein C (and protein S), which, conversely, are not affected by NOACs. As a consequence, VKAs disrupt, while NOACs preserve, protein C activities (Figure 1).

VKAs also interfere with the synthesis of non-coagulation proteins that require vitamin K as a cofactor. In particular, VKAs impede vitamin K-dependent carboxylation of gamma-carboxyglutamic acid (Gla) of matrix Gla protein (MGP), which inhibits vascular calcification [42]. As a consequence, vessel calcification is enhanced.

Therefore, NOACs are expected to not perturb, or at least to perturb less, the homeostasis of the CV system, and to antagonize at least some vascular disease processes.

Several lines of experimental research buttress this hypothesis. Blockade of FXa-PAR signaling was shown to counteract atherogenesis [43,44] and diabetic vasculopathy [45], and inhibition of thrombin-PAR-1 was found to halt the development and progression of AF by preventing the molecular and cellular changes that serve as a substrate for the arrhythmia [46,47,48].

The demonstration of the advantage of targeting PAR effects with NOACs in patients is still lacking. Nonetheless, indirect evidence does relate FXa with atherosclerotic burden [44], and the reduction in atherosclerotic events observed in dedicated trials with NOACs, as detailed below, lends further support to the concept that NOACs may have pleiotropic, favorable actions in the CV system, extending beyond anticoagulation. Studies in patients also demonstrated that VKA users developed significantly more calcified coronary plaques as compared to VKA non-users [49].

## 3. Clinical Considerations Supporting the Switch from Vitamin K Antagonists to Non-Vitamin K Antagonists

### 3.1. Decreased Intracranial Hemorrhage

ICH is a major life threat, with mortality estimated at about 40% at one month and increasing to about 60% at one year [50]. Overall, the incidence has been found to be 24.6 per 100,000 person-years, and similar in men and women.

ICH can be intraparenchymal—referring to nontraumatic bleeding into the brain parenchyma; subarachnoid—referring to bleeding into the space between the pia and the arachnoid membranes occurring because of rupture of cerebral aneurysms, bleeding from arteriovenous malformations or tumors, cerebral amyloid angiopathy, and vasculopathies; subdural hematoma—due to bleeding between the dura and the arachnoid; and epidural hematoma—involving bleeding between the dura and the bone. Subdural and epidural hematomas are usually traumatic.

Age is the main risk factor for ICH, with incidence ratios increasing from 0.10 per 100 patient-years for people aged less than 45 years to 9.6 for people older than 85 years [50]. Other common risk factors for spontaneous ICH are hypertension and age- and hypertension-related cerebral amyloid angiopathy, related to the build-up of amyloid proteins in arterial walls, making them more susceptible to rupture. The most important modifiable risk factors are, however, antithrombotic treatments, hypertension, tobacco, and cocaine use [51]. Among these, antithrombotic treatments have a major role. Even antiplatelet medications, such as aspirin and clopidogrel, when used alone, appear to increase the rate of ICH by about 30–40% [52]. Combination of clopidogrel and aspirin has a higher rate of ICH compared with clopidogrel alone [53] and aspirin alone [54]. Antiplatelet agents used alone, however, are associated with a lower rate of ICH compared with VKAs [55]. Among anticoagulants, heparin (unfractionated or low molecular weight heparin, LMWH), VKAs, fibrinolytic agents (e.g., alteplase, tenecteplase) may all lead to ICH. Warfarin and other VKAs strongly contribute, in epidemiological terms, to the risk of ICH, with important evidence suggesting that even a perfectly conducted VKA treatment, with INR between 2.0 and 3.0 in AF patients, doubles the risk of ICH [56]. Adding antiplatelet therapy to warfarin further increases the risk of ICH [57]. Factors modifying the risks are the underlying reason for therapy, dosage, and the control in its use. Anticoagulant prophylaxis with minidose heparin (5000 units subcutaneously twice daily) and LMWH, conversely, appear to be safer, and these regimens are indeed used as prophylaxis of venous thromboembolism even a few days after ICH [58].

Because of these data, for long, a widely held belief is that ICH is an inevitable drawback of any therapeutic anticoagulation, and VKAs, in particular. The dogma was however challenged with publications, one after the other, of the four pivotal trials of the NOACs—dabigatran [59], rivaroxaban [60], apixaban [61], and edoxaban [62]. All these trials showed a significantly lower rate of ICH in the NOAC arm. Such data have been confirmed in large registries, such as PREFER in AF/PREFER Prolongation [63,64], GARFIELD [65], and ETNA-AF [66].

**Figure 3:**
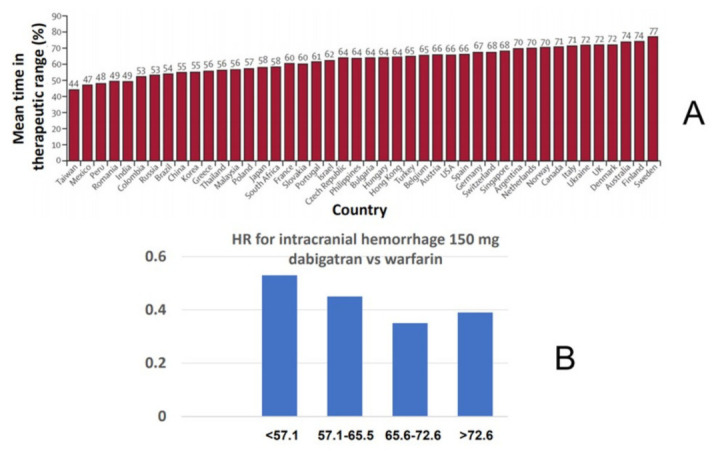
Relative risk of intracranial hemorrhage for dabigatran vs. warfarin as a function of time in therapeutic range (cTTR) of centers participating in the RE-LY trial. (**A**) Distribution of mean cTTR in countries participating in the trial. (**B**) Hazard ratios (HR) for intracranial hemorrhage in the comparison of dabigatran 150 mg twice daily vs. warfarin as a function of cTTR.

At variance from major bleeding, the lower rate of ICH with the NOACs compared with warfarin appears to be independent of the quality of anticoagulation with warfarin. Indeed, in the RE-LY trial, the hazard ratios for ICH for both dabigatran doses vs. warfarin were always <1 when stratified by countries participating in the study with quite varying center TTR [24] (Figure 3). A similar analysis has also been performed with rivaroxaban in the ROCKET-AF trial [26]. Thus, even in conditions of “optimal” warfarin anticoagulation control, NOACs appear to confer a net safety advantage with regard to ICH, consistent with the notion that most cases of ICH appear while the INR is within the recommended range [67].

Reasons why NOACs lead to a lower rate of ICH are still largely speculative, but at least in part appear to be due to the lack of interference by NOACs with the tissue factor-FXa pathway, which is an important factor protecting from cerebral bleeding, and curtailed by the decreased concentrations of FVIIa in the plasma of VKA-treated patients [68] (Figure 1). Whatever the underlying reason, a much lower risk of ICH is an important—possibly the most important reason—not only to start anticoagulation therapy, when indicated, with a NOAC instead of a VKA; but also, to switch to a much safer NOAC even in a well-controlled VKA-treated patient.

### 3.2. Renoprotection

Incidence of both chronic kidney disease (CKD) and AF sharply increases with age determining tremendous overlap with serious consequences as CKD increases the risk of both thromboembolic stroke and major bleeding [69]. In a large population of 85,116 patients with incident AF, those with impaired renal function were older and had more comorbidities. Higher CKD stages were associated with worse outcomes. Stroke rates increased from 1.04 events per 100 person-years in stage 1 CKD to 3.72 in stages 4–5 CKD. Mortality increased from 3.42 to 32.95 events/100 person-years, and bleeding rates increased from 0.89 to 4.91 events/100 person-years [70].

Thus, maintaining adequate renal function is particularly desirable in patients with AF. Unfortunately, renal function decline is very common among those patients, particularly when treated with VKAs. In a real-life study including consecutive stable anticoagulated patients with AF (warfarin with INR 2.0–3.0 in the previous six months), the changes in renal function during a long-term follow-up period were assessed. The mean estimated glomerular filtration rate (eGFR) in this cohort decreased >10 mL/min/1.73 m^2^ in 21% of the patients during a 2-year follow-up. The variables associated with severe renal impairment during follow-up were heart failure (HR 3.58, 95% CI 1.36–9.42), basal eGFR (HR 6.34, 95% CI 2.44–16.50), and CHADS2 score (HR 1.63, 95% CI 1.19–2.23). A low eGFR was associated with thrombotic events, with every 30 mL/min/1.73 m^2^ eGFR decrease (HR 1.42, 95% CI 1.11–1.83), bleeding (HR 1.44, 95% CI 1.08–1.94), and mortality (HR 1.47, 95% CI 1.13–1.91) [71].

VKAs are potentially nephrotoxic. Although most animal models suggest that VKAs are nephrotoxic due to their anticoagulant effects, which can cause glomerular hemorrhage with subsequent renal tubular obstruction and tubular epithelial injury [43,72,73], they may also induce renal damage by increasing vascular calcification.

Moreover, some warfarin-treated patients experienced an accelerated progression of CKD [74] and acute kidney disease associated with excessive anticoagulation [75]. Acute kidney injury (AKI) in warfarin-treated patients with CKD may occur shortly after an acute increase in the INR >3.0 with the formation of occlusive red blood casts. Recovery from this warfarin-associated AKI is poor since subsequent CKD progression is accelerated [76]. Declining kidney function is a critical determinant of unfavorable outcomes in patients with AF and CKD. In a large primary care UK electronic database, 18,240 patients with stage 3/4 CKD were prospectively followed for a median follow-up of 3.2 years. An accelerated decline in kidney function strongly predicted for higher risk of major bleeding (HR 1.09 per mL/min/1.73 m^2^/year increase in eGFR decline), hospitalization (HR 1.06), and death-from-any-cause (HR 1.11), but not for stroke/systemic embolism (HR 0.97) [77].

The evidence showing benefits with the use of VKAs, and the lack of available alternatives have outweighed the potential of adverse effects from those compounds. However, with the availability of NOACs as alternatives, understanding the relative risks of nephrotoxicity of VKAs has become important.

Large RCTs comparing NOACs and VKAs for stroke prevention have provided an opportunity to compare these agents’ effects on renal function.

The RE-LY and the ROCKET-AF trials comparing dabigatran or rivaroxaban to warfarin, respectively, reported that the rate of decline of renal function was slower in patients allocated to dabigatran or rivaroxaban than in those allocated to warfarin (eGFR decline of 0.98 mL/min/year with dabigatran compared to 1.47 mL/min/year with warfarin; and mean 3.5 mL/min loss of eGFR in rivaroxaban-treated patients compared to 4.3 mL/min loss in warfarin-treated patients) [78,79]. The ARISTOTLE trial, however, comparing apixaban to warfarin, showed a small but greater decline in kidney function in apixaban-treated patients (mean 0.41 mL/min after 12 months, *p* = 0.01) [80].

These analyses were all post-hoc and not pre-specified and, although suggestive of the potential for nephrotoxicity from VKA, there is still considerable uncertainty; especially in light of divergent findings from the three trials.

**Figure 4:**
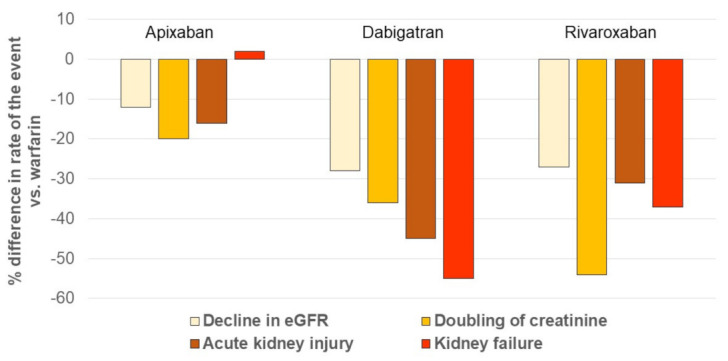
Incidence of renal outcomes in patients with AF receiving NOACs, compared with the one in patients taking warfarin. The figure was drawn based on the published data in reference [82]. AKI: acute kidney injury, defined as hospitalization or emergency department visit with a primary or secondary diagnosis code of AKI; eGFR: estimated glomerular filtration rate. Kidney failure was defined as eGFR lower than 15 mL/min per 1.73 m^2^, having a kidney transplant, or undergoing long-term dialysis.

In a real-world study, using a large (9769 patients) US administrative database and high-quality methods for comparative effectiveness research, dabigatran and rivaroxaban were independently associated with a lower incidence of both acute and chronic renal events compared to warfarin, while apixaban was not (Figure 4).

When the three NOACs were pooled, the relative effects were a 23% reduced risk of decline in eGFR, a 38% reduction in doubling serum creatinine, and a 32% reduction in AKI compared with warfarin [81].

The findings in the study of Yao et al. broadly agree with the findings in the RCTs, including the lack of benefit from the use of apixaban compared to warfarin.

Noteworthy, experimental investigations indicate that NOACs may also be nephroprotective by abrogating PAR signaling. For instance, it was recently reported that FXa and PAR-2 exacerbate, and edoxaban ameliorates, diabetic nephropathy through the modulation of inflammation [82].

In summary, data from both RCTs and real life provide a piece of evidence that helps clinicians in weighing the benefits and risk of NOACs versus warfarin, since at least some NOACs are associated with less nephrotoxicity. This is of particular importance because some kidney outcomes (e.g., decline in eGFR or acute kidney injury) are often more common than stroke or major bleeding.

There remains uncertainty over whether such effects are similar between all NOACs. The selection of the most appropriate OAC can be a complex, multistep process that is based on the consideration of several clinical variables including, importantly, the assessment of renal function [83]. Mainly for patients at high risk of progressive loss of kidney function who do not have a contraindication to a NOAC, the putative renal benefits are one more reason to choose a NOAC over VKA [84].

However, it needs to be considered that there are no outcome data for NOACs in patients with advanced CKD (creatinine clearance <30 mL/min), and there are conflicting data regarding the benefits and risks of these medications in end-stage renal disease (creatinine clearance <15 mL/min) [85]. At present, the alternatives to VKA in those CKD stages are limited to left atrial appendage occlusion, antiplatelet therapy, or even no anticoagulation [86].

### 3.3. Reduction of Cardiovascular Events

Patients with AF are at heightened risk of ischemic CV events as compared with subjects without AF, owing to shared risk factors and common pathogenetic pathways [87,88]. In this regard, it has been speculated that NOACs may provide protection against major adverse CV events not limited to stroke and SE associated with AF [40].

This paradigm has gained traction in recent years, after the publication of the results of the COMPASS trial, in which 27,395 patients with coronary artery disease and/or peripheral artery disease were randomized to aspirin, rivaroxaban 5 mg b.i.d., or the combination of rivaroxaban 2.5 mg b.i.d. and aspirin [89]. The latter intervention reduced the primary composite endpoint of CV death, myocardial infarction, or stroke by 24% as compared with aspirin alone, and all-cause mortality by 18%. Major bleeding was instead more frequent with dual antithrombotic therapy than with aspirin, although the rate of fatal or critical bleeding was not significantly different between the two arms [89]. These results were confirmed in the prespecified subgroups of participants with peripheral artery disease [90] and coronary artery disease [91].

Important caveats must be raised while discussing the COMPASS data. First, rivaroxaban 5 mg b.i.d. alone was not better than aspirin alone with respect to the primary outcome, while significantly increasing the risk of major bleeding [89,90,91]. Thus, FXa inhibition on top of single antiplatelet therapy, rather than FXa inhibition alone, appears to be superior to single antiplatelet therapy to diminish the incidence of CV events in patients with atherosclerotic disease. Moreover, the dosage of rivaroxaban tested was different from the one approved for the prophylaxis of cardioembolism in AF. It should be also noted that prior studies evaluating the efficacy of NOACs on cardiovascular events, such as myocardial infarction, yielded discordant results [92].

On the other hand, the CV effects and, thereby, the benefit of NOACs may be wider than previously assumed. Recent advances in understanding the mechanisms underlying peripheral vascular events are consistent with this view. Indeed, it was shown that thrombotic occlusion without significant atherosclerosis is often the cause of critical limb ischemia, especially of infra-popliteal arteries, and that the distal small vessels in amputation specimens for critical limb ischemia are characterized by luminal thrombi, cholesterol emboli, intimal fibrosis, and medial calcification [93]. It is also noteworthy that in the real-world setting, with an overall incidence of myocardial infarction higher than in RCT, the risk of MI appeared to be lower with NOACs than VKAs [94].

## 4. Additional Evidence That May Reinforce the Choice of Using Non-Vitamin K Antagonists Instead of Vitamin K Antagonists

Ongoing research into NOACs is expected to produce further data supporting the switch from VKAs to NOACs. Patients’ preferences are critical in determining adherence to OAC. In a recent study from Asia, the substitution of VKA therapy with dabigatran was associated with a significant improvement in treatment convenience and satisfaction [95], suggesting that NOACs are favored by AF patients themselves. Patients’ socio-demographic characteristics and physicians’ level of experience with VKAs vs. NOACs are other factors that could be relevant in choosing the type of OAC [18]. Furthermore, costs and reimbursement criteria may be important and underlie significant dissimilarities in OAC prescription patterns across different healthcare systems. Finally, the results of ongoing clinical trials (NCT02618577 and NCT01938248) are awaited to conclude whether NOAC therapy is indicated in patients with device-detected subclinical AF.

## 5. Conclusions

Both pharmacological and clinical argumentations warrant the switch from VKAs to NOACs irrespective of TTR being low or high. We propose to systematically substitute NOACs for VKAs in patients with AF.

## Data Availability

Not applicable.
